# Clinical Effects of Microwave Ablation in the Treatment of Low-Risk Papillary Thyroid Microcarcinomas and Related Histopathological Changes

**DOI:** 10.3389/fendo.2021.751213

**Published:** 2021-09-16

**Authors:** Chenya Lu, Xingjia Li, Xiaoqiu Chu, Ruiping Li, Jie Li, Jianhua Wang, Yalin Wang, Yang Xu, Guofang Chen, Shuhang Xu, Chao Liu

**Affiliations:** ^1^Endocrine and Diabetes Center, Affiliated Hospital of Integrated Traditional Chinese and Western Medicine, Nanjing University of Chinese Medicine, Jiangsu Province Academy of Traditional Chinese Medicine, Nanjing, China; ^2^Key Laboratory of TCM Syndrome & Treatment of Yingbing of State Administration of Traditional Chinese Medicine, Jiangsu Province Academy of Traditional Chinese Medicine, Nanjing, China; ^3^Department of Pathology, Affiliated Hospital of Integrated Traditional Chinese and Western Medicine, Nanjing University of Chinese Medicine, Jiangsu Province Academy of Traditional Chinese Medicine, Nanjing, China; ^4^Department of Ultrasound, Affiliated Hospital of Integrated Traditional Chinese and Western Medicine, Nanjing University of Chinese Medicine, Jiangsu Province Academy of Traditional Chinese Medicine, Nanjing, China; ^5^Department of General Surgery, Affiliated Hospital of Integrated Traditional Chinese and Western Medicine, Nanjing University of Chinese Medicine, Jiangsu Province Academy of Traditional Chinese Medicine, Nanjing, China

**Keywords:** papillary thyroid microcarcinoma, microwave ablation, thermal ablation, pathological changes, papillary thyroid carcinoma

## Abstract

**Objective:**

This study aimed to evaluate the feasibility and efficacy of ultrasound-guided percutaneous microwave ablation (MWA) in the treatment of low-risk papillary thyroid microcarcinoma (PTMC), and to observe the histopathological changes after MWA.

**Methods:**

MWA was performed under ultrasound guidance for 73 unifocal PTMC patients without clinically cervical or distant metastasis. The target ablation zone exceeded the tumor edge (judged by contrast-enhanced US) to avoid marginal residue and recurrence. Ultrasound evaluation was performed at 1 day, 1, 3, 6, 12 and 24 months after treatment, and thyroid function evaluation at the first 6 months. Repeated fine needle aspiration cytology or core needle biopsy pathology was performed at 3 or 6 months after MWA to evaluate residual tumors. Any adverse event associated with MWA was evaluated.

**Results:**

The follow-up after MWA lasted 6 (6, 12) months. Tumor volume decreased significantly from 0.06 mm^3^ (0.04, 0.11 mm^3^) to 0.03 mm^3^ (0.00, 0.06 mm^3^) at 12 months after MWA (*P*< 0.001), with a median volume reduction ratio of 80.28% (-7.43, 100%) and 16 cases (21.92%) presenting complete remission. The largest diameter, volume and ablation energy were found to be different in patients with and without complete remission 12 months after MWA. On histopathological examinations, no atypical or malignant follicular cells were identified after thermal ablation. The most common pathological characteristics were fibroblastic proliferation (34/39, 87.18%) and chronic inflammation (32/39, 82.05%), followed by infarction (21/39, 53.85%). Five patients were transferred to thyroidectomy and 4 of them were confirmed with local recurrence and/or lymph node metastasis. Serum thyrotropin decreased transiently after MWA (*P*< 0.01) but normalized thereafter. No serious and permanent complications were reported.

**Conclusions:**

MWA is a safe and effective treatment for low-risk PTMC. Fibroblastic proliferation and chronic inflammation are the most common pathological changes after MWA of PTMC.

## Introduction

Thyroid carcinoma has seen an incidence rising the fastest recently. Its major subtype is papillary thyroid microcarcinoma (PTMC) with a maximum diameter of 1.0 cm (1). Conventional surgical procedures are preferrable for thyroid carcinoma. In Japan, South Korea and the United States, some low-risk PTMC cases are managed by active surveillance, rather than an immediate surgery. Interestingly, most low-risk PTMC maintains stable, only progressive in rare cases (2). Hence, it is feasible to perform microwave ablation (MWA) in low-risk PTMC cases. Although its application is doubted by potential tumor residues and lymphatic metastasis, MWA still exhibits higher safety and less trauma in its limited treatment of low-risk PTMC cases in China. In the present study, a total of 73 PTMC patients were treated with MWA under ultrasound guidance, and regularly followed up for re-examinations. We analyzed the efficacy and safety of MWA for PTMC, and postoperative morphology by core needle biopsy (CNB).

## Materials and Methods

### Study Oversight

The study protocol was approved by the ethics committee of Affiliated Hospital of Integrated Traditional Chinese and Western Medicine, Nanjing University of Chinese Medicine. All subjects were diagnosed with PTMC by thyroid fine-needle aspiration cytology (FNA) or pathology by CNB. All the patients were clearly informed that MWA could not fully avert the risk of recurrence, lymph node and distant metastasis. Written informed consent was obtained from all study participants.

### Patients

In this retrospective single-center study, 73 patients (13 males, 60 females) had pathologically diagnosed PTMC and received MWA from December 2017 to June 2021 at our hospital. Their ages ranged from 16 to 74 years, with an average of 38.71 ± 11.82 years.

The inclusion criteria: (i) a diagnosis of papillary thyroid carcinoma (PTC) by FNA or CNB; (ii) a single PTC that was no more than 10 mm in greatest diameter; (iii) no invasion into tissues surrounding the thyroid gland on ultrasound; (iv) no suspicious or malignant cervical lymph nodes or distant metastasis; and (v) patient with intolerance to surgery or refusal to both surgery and active surveillance after being fully informed.

The exclusion criteria: (i) other types of thyroid cancer; (ii) allergic to the drugs used in the study; (iii) severe bleeding tendency or coagulation dysfunction; (iv) immunosuppressive drugs was required during the trial; (v) infection, high fever or leukocyte abnormality; (vi) severe heart, respiratory, liver disease or renal failure; (vii) pregnancy and lactation. All patients were prohibited from taking antiplatelet or anticoagulant medications for at least 1 week preoperatively.

### MWA System

The MWA system (KY-2000) used in this study was produced by Nanjing Kangyou Co., Ltd. (Jiangsu, China). A cooled-tip needle for MWA was used for the treatment of the thyroid nodule; the needle was 1.6 mm in diameter, and the handle was 10 cm in length. The distance between the electrode and the needle tip was 3 mm.

### US System

The Siemens Acuson S2000 (Siemens Mountainview, USA) ultrasound diagnostic instruments were used for recording images and guiding the MWA procedure.

### Pre-Ablation Assessment

Senior ultrasonographers collected the pre-ablation ultrasonography data. All thyroid nodules were scanned from multiple angles across sections. Information on size, volume (V = πabc/6), calcification, blood flow distribution, location, and surrounding tissues was recorded to determine an optimal approach for MWA. All lesions were confirmed as PTMC by FNA or CNB.

### MWA Procedure

All US-guided procedures were performed by one experienced doctor. The patient was placed in a supine position with the neck hyperextended. The skin was sterilized and draped. Thyroid MWA was performed under local anesthesia (1.0% lidocaine) guided by US. Normal saline was carefully injected into the thyroid capsule as hydrodissection to protect major structures (carotid vessels, trachea, recurrent laryngeal nerve, esophagus) from thermal injury.

With the guidance of real-time US imaging, a microwave antenna was inserted into the thyroid to ablate the PTMC lesions according to the predetermined strategy. MWA was performed using the multidimensionally fixed needle ablation technique. A 16-gauge ablation needle was inserted into the tumor under US guidance. The tumor and adjacent thyroid gland were included in the ablated zone (2-5 mm) to prevent marginal recurrence. A power output of 25-35 W at 2450 MHz was routinely used, inducing coagulation necrosis in the tumor. The ablated time was for 38-467s. The operation was sustained until the entire tumor was hyperechoic. When withdrawing the antenna, the needle track was coagulated to prevent tumor cell seeding. We spoke with the patients intermittently during the entire procedure to monitor their vocal status. A contrast-enhanced ultrasound (CEUS) was repeated to evaluate the ablated area after MWA.

### Follow-Up

Precise ultrasound examination was performed at 1, 3, 6, 12, and 24 months after MWA and every year thereafter. The size and volume reductions of the ablation area and cervical lymph nodes were observed and recorded. The volume reduction rate (VRR) of the lesion was calculated by VRR(%) = ([initial volume–final volume]×100%)/initial volume. In cases presenting a “black line” for the ablation area that healed, the volume could not be calculated. We defined this situation as completely ablated as well. The thyroid function was retested at the first half year after MWA, and no patients received thyroid hormone suppression therapy after MWA. CNB or FNAC was performed at 6 months after MWA.

Tumor progression was defined according to the following two criteria: (i) new PTC confirmed by FNA or CNB; (ii) cervical or lateral lymph node metastasis confirmed by FNA, CNB, or thyroglobulin washout concentration. After ablation, patients routinely underwent neck and lung CT at 12 months after MWA to detect recurrence and distal metastasis. However, for patients with symptoms or serious concerns regarding metastasis, 18F-fluorodeoxyglucose positron emission tomography CT or a bone scan was considered.

### Statistical Analysis

SPSS 22.0 was applied for statistical analysis. Data in normal distribution were presented as the mean ± standard deviation and compared by *t*-test. Data not in normal distribution were expressed by medians and quartiles and analyzed through nonparametric test. A p-value of <0.05 was considered statistically significant.

## Results

### Clinical Characteristics

Each of the 73 enrolled subjects was confirmed with solitary PTMC by FNA or CNB. Their average age was 38.71 ± 11.82 years. Of all the nodules, 33 and 40 were in the left and right lobe, respectively. The largest diameter was 0.58 ± 0.16 cm (0.24-1.00 cm). The median volume was 0.06 cm^3^ (0.04, 0.11) cm^3^ (**Table 1**). In the MWA procedure, the median ablation time was 80.50 (58.50, 121.50) seconds, and the median ablation energy was 2310.00 (1587.50, 3675.00) Joule. Contrast-enhanced US revealed that the complete ablation rate of the nodules was 100%.

**Table 1:** Volume change at ablated areas.

Follow-up	Pre-ablation	1 day	1 month	3 months	6 months	12 months	24 months
(n)	(73)	(73)	(64)	(53)	(52)	(34)	(9)
Volume (cm^3^)	0.06 (0.04, 0.11)	1.58 (0.97, 2.25)	0.79 (0.50, 1.10)	0.36 (0.17, 0.54)	0.08 (0.03, 0.18)	0.03 (0.00, 0.06)	0.00 (0.00, 0.00)
*Z*		7.424	6.948	6.069	2.085	-2.368	-2.666
*P*		0.000	0.000	0.000	0.037	0.018	0.008
VRR (%)		-2867.98 (-5257.14, -933.31)	-1226.35 (-2083.30, -614.80)	-344.42 (-753.54, -134.40)	-29.66 (-177.45, 69.54)	80.28 (-7.43, 100.00)	100.00 (100.00, 100.00)

### Follow-Up

**Figure 1:**
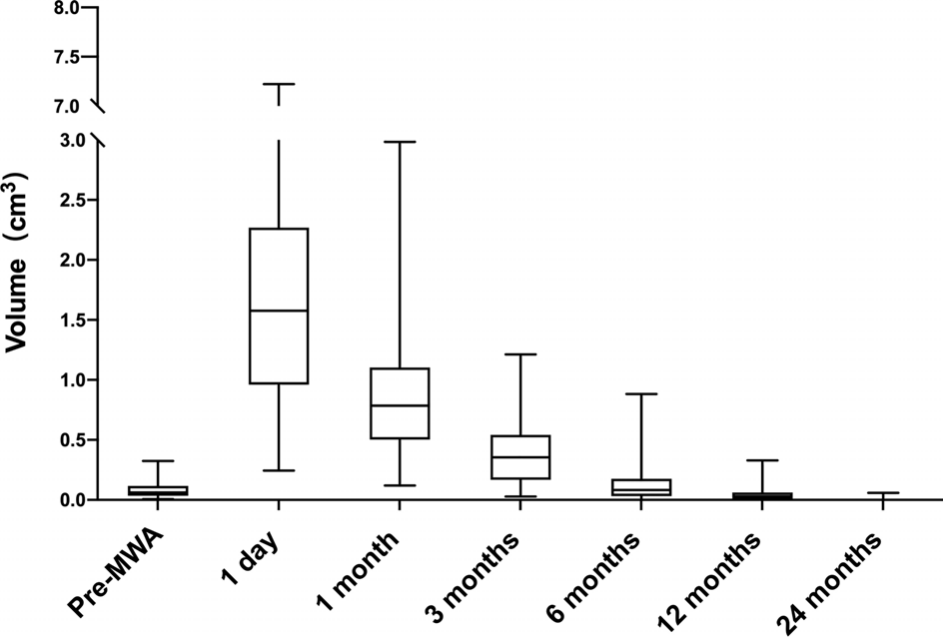
Volume change of ablated areas (baseline and after 1, 3, 6, 12 and 24 months from the microwave ablation). The procedures significantly increased volume at one month (p < 0.001 *vs.* baseline). Subsequently, the tendency of volume is to shrink. The volume was decreased at 12 months (*P* < 0.05 *vs.* baseline).

The median follow-up duration was 6 (6, 12) months. The median pre-ablation volume of the nodule and post-ablation area at each follow-up time point are summarized in **Table 1**. The median pre-ablation volume was 0.06 cm^3^ (0.04, 0.11 cm^3^). As the ablation margin was extended, the median area was significantly enlarged to 1.58 cm^3^ (0.97, 2.25 cm^3^) (p < 0.001) at 1 day after ablation. Thereafter, the volume significantly decreased at 12 months of follow-up (p = 0.046), with a median volume of 0.03 cm^3^ (0.00, 0.06 cm^3^) (**Table 1**). The VRR was 100.00% (100, 100%) at 24 months of follow-up. The volumes at each follow-up time point are shown in **Figure 1**.

**Figure 2:**
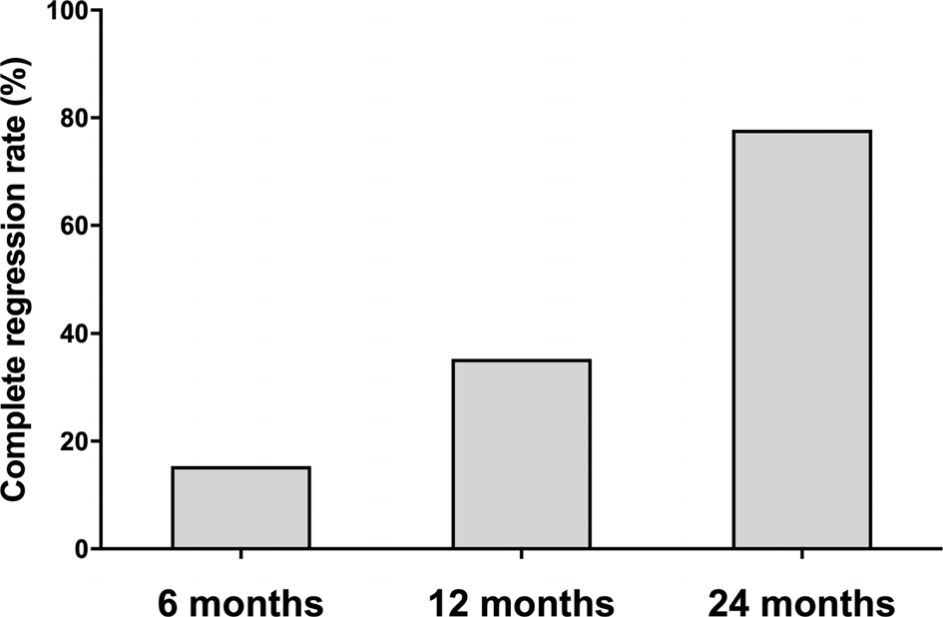
The complete regression rate at each follow-up point. The complete regression rate increased during 24-month follow-up. The number of absorbed ablation zone was 8/52, 12/34 and 7/9 patients at 6, 12, and 24 months follow-up.

Of the 73 ablated nodules, 16 were ablated with a complete regression rate of 21.92%. The complete regression rates were 15.38%, 35.29% and 77.78% at 6, 12 and 24 months of follow-up, separately (**Figure 2**). Of all the 34 patients, 12 ablated PTMC disappeared at 12 months of follow-up. The Largest diameter, volume and ablation energy were found to be different between patients whose ablation areas were totally absorbed and those not at 12 months after MWA (**Table 2**).

**Table 2:** Comparison of characteristics between patients with and without complete remission at 12 months follow up.

	Disappearance	Appearance	*t/Z*	*P*
Number	12	22		
Age (years)	34.46 ± 9.57	39.32 ± 14.53	-1.072	0.291
Largest diameter (cm)	0.46 ± 0.14	0.59 ± 0.15	-2.426	0.021
Volume (cm^3^)	0.04 (0.01, 0.04)	0.11 (0.05, 0.12)	2.799	0.004
Distance from the nearest capsule (cm)	0.07 ± 0.08	0.11 ± 0.10	-1.299	0.203
Distance from the dorsal capsule (cm)	0.31 ± 0.30	0.26 ± 0.22	0.546	0.589
Ablated time (s)	63.00 (45.00, 104.00)	70.50 (45.00, 82.00)	-0.017	0.987
Ablated volume (cm^3^)	1.22 ± 0.75	1.55 ± 0.96	-1.060	0.297
Energy (J)	1650.00 (1350.00, 3120.00)	2115.00 (1350.00, 2460.00)	0.068	0.960
Energy/volume (J/ml)	89825.25 (32553.32, 134024.22)	23213.86 (12570.99, 43798.03)	-2.629	0.008
TSH (µIU/ml)	1.97 (1.36, 3.04)	1.59 (1.29, 2.94)	-0.656	0.529
FT4 (pmol/l)	16.80 ± 2.22	16.01 ± 2.59	0.907	0.371
FT3 (pmol/l)	4.82 ± 0.49	4.86 ± 0.48	-0.246	0.808

None of the patients received thyroid-stimulating hormone (TSH) suppressive therapy during the follow-up. Before MWA, eight patients took levothyroxine for hypothyroidism caused by thyroidectomy or thyroiditis. One patient increased levothyroxine dosage after MWA. As shown in **Table 3**, TSH (*P*=0.000) significantly decreased, while free thyroxine (FT4, *P*= 0.000), free triiodothyronine (FT3, *P*= 0.000), and thyroglobulin (Tg, *P*=0.000) were significantly increased at 1 day after MWA. However, thyroid function quickly returned to preoperative levels at 1 month follow-up. During the 6 months of follow-up, only two patients (33.33%) developed subclinical hypothyroidism, with positive TgAb or TPOAb under enhanced CT before ablation.

**Table 3:** Changes in thyroid function after MWA.

Follow up	Number	TSH (μIU/ml)	FT4 (pmol/L)	FT3 (pmol/L)	Tg (ng/ml)	TgAb(IU/ml)
Pre-MWA	62	2.00 (1.31, 3.20)	16.30 (14.20, 17.96)	4.70 (4.33, 4.96)	7.74 (3.71, 14.98)	11.97 (10.00, 48.44)
1 day after MWA	65	0.69 (0.54, 1.03)**	22.22 (19.99, 25.64)**	5.21 (4.56, 5.92)**	495.70 (257.08, 500.00)**	12.45 (10.00, 16.02)**
1 month after MWA	54	2.02 (1.51, 2.78)	15.55 (14.08, 17.39)	4.84 (4.35, 5.21)	8.19 (2.53, 11.88)	12.63 (10.00, 78.94)**
3 months after MWA	44	2.30 (1.76, 3.43)	16.05 (14.77, 17.96)	4.81 (4.41, 5.05)	6.80 (3.51, 16.38)	12.35 (10.00, 50.58)
6 months after MWA	38	2.77 (1.70, 3.58)	15.88 (14.07, 17.61)	4.82 (4.33, 5.09)	7.18 (3.42, 12.32)	13.50 (10.59, 59.61)

**Compared with pre-MWA, P < 0.01.

During the follow-up period, five patients received thyroidectomy. Four of them were found to have new suspicious nodules or lymph nodes on routine US examination, and diagnosed with recurrence by FNA. The postoperative pathologies confirmed one local recurrence, one lymph node metastasis and two local recurrences combined lymph node metastasis. No distant metastasis was observed.

### Pathological Changes

**Figure 3:**
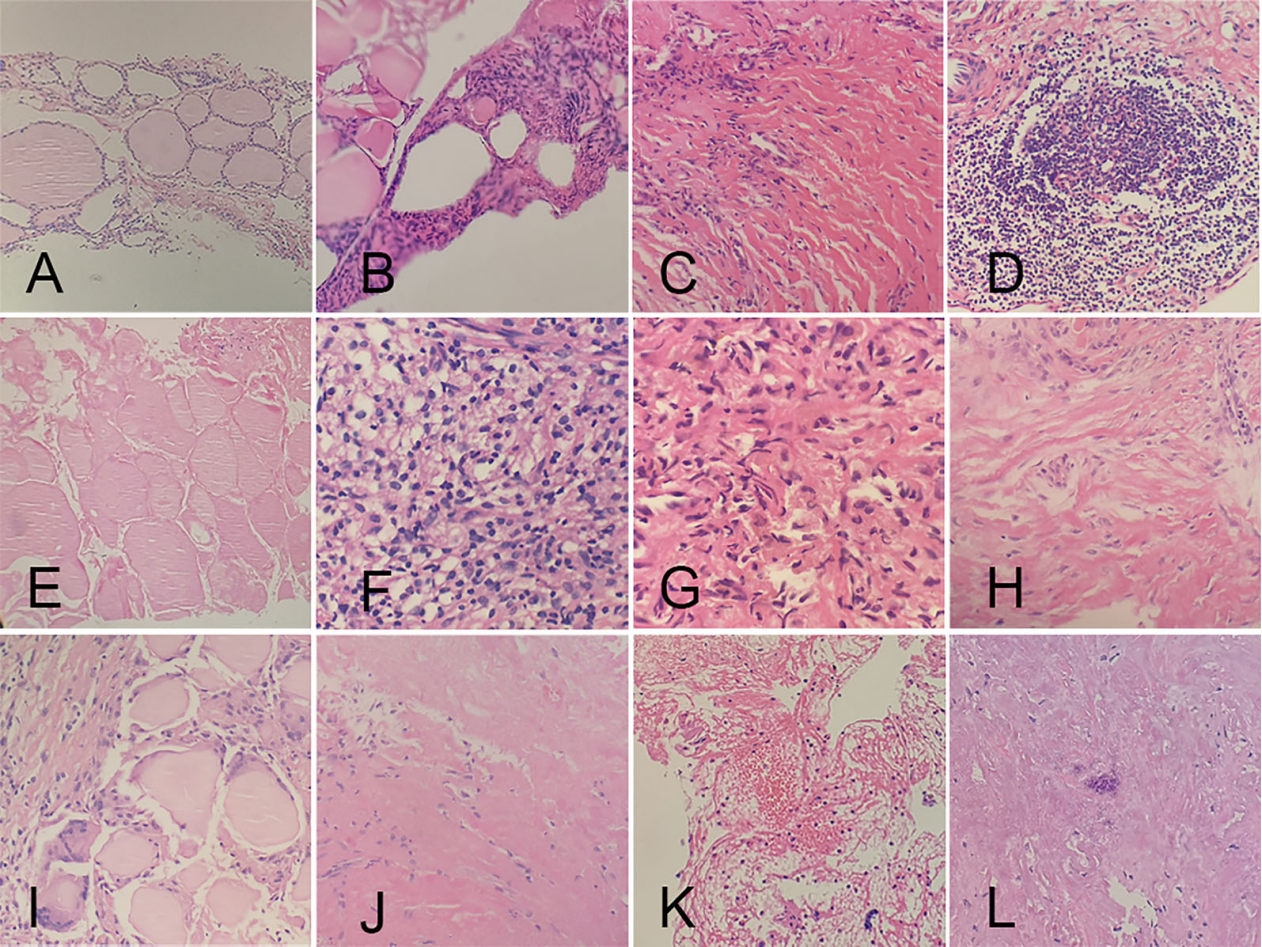
Histopathologic characteristics of ablated areas after microwave ablation. **(A)** follicular cells; **(B)** atypical follicular cells; **(C)** fibroblastic proliferation; **(D)** chronic inflammation; **(E)** infarction; **(F)** histiocytic deposition; **(G)** blackish particle; **(H)** foreign body reaction; **(I)** cholesterol granuloma; **(J)** acellular hyalinization; **(K)** hemorrhage; **(L)** calcification.

CNB and FNA were performed in 42 and 2 patients, respectively. Twenty received CNB at 3 months, while the others at 6 months of follow-up. No characteristics of PTC were found in all the patients. On histopathological examinations, fibroblastic proliferation was found in 87.18% (34/39), followed by chronic inflammation in 82.05% (32/39). Chronic inflammation was diffuse in 9.38% (3/32). The rest pathological changes had a high-to-low order of prevalence as follows: infarction (21/39, 53.85%), histiocytic deposition (9/39, 23.08%), blackish particle (7/39, 17.95%), foreign body reaction (7/39, 17.95%), cholesterol granuloma (7/39, 17.95%), acellular hyalinization (4/39, 10.26%), hemorrhage (1/39, 2.56%), and calcification (1/39, 2.56%) (**Figure 3**). The procedure had neither altered the thyroid capsule nor changed the thyroid tissue adjacent to the treated area, with maintained follicular architecture and benign cytological characteristics.

### Complications

Of the 73 enrolled subjects, no intolerable pain or obvious discomfort that required suspension of the treatment occurred during the ablation. Hoarseness occurred to one patient after ablation and resolved three months later. No skin burns, or infection occurred after ablation, and no analgesics were needed. Two patients developed sub clinical hypothyroidism without medical treatment until this article is completed.

## Discussion

Active surveillance or thermal ablation, rather than immediate surgery, has been highlighted as a non-conventional treatment of very low-risk PTMC by various institutions (3, 4). Indications and contraindications of active surveillance have been identified in China (5), although its clinical application is being debated (6). The efficacy of MWA in treating PTMC has been confirmed in several studies, but also showing differences unexplained yet. The histological changes of thyroid tissue after microwave ablation for papillary thyroid carcinoma have not been reported yet.

Our findings verified that MWA was effective in the treatment of PTMC. Typical characteristics of PTC were not identified by FNAC or CNB in the third or sixth month of follow-up, suggesting the reliable efficacy of MWA. During the 2-year follow-up, we detected 4 recurrences with a recurrence rate of 5.48%, including local recurrence and lymph node metastasis. The recurrence rate reported by other centers was 0.5%-4.2% (7–9). It suggested the importance of preoperative assessment and early detection of recurrence. Postoperative CNB and FNA at 3 or 6 months of follow-up were reported as effective tools for screening tumor recurrence after thermal ablation (10). In our study, the recurrence was suspected by routine ultrasound examination and then confirmed by FNA, and none had been detected by the routine FNA or CNB *in situ*. Therefore, relatively active surveillance with careful ultrasound examination during follow-up is recommended to detect the early recurrence.

Our study showed a complete absorption rate of 21.92%, and the disappearance rate of ablation lesions increased with the prolongation of follow-up. A previous review showed great differences in the absorption rate after MWA in different trials, from 15.2% to 97.6% (11). In present study, maximum diameter, baseline volume and ablation energy were factors of complete absorption. In a Korean cohort, nodules with a perioperative diameter < 5 mm were more likely to disappear than those with a maximum diameter of 5 mm (12).

Volume reduction of PTMC following ablation differs from that of benign nodules (13). The volume of ablated lesions rapidly increases in a short period, then immediately shrinks, followed by a slow shrinkage or even disappearance. The volume reduction is closely linked to the procedures of extended ablation. An Italia research on thermal ablation of benign thyroid nodules showed that the ablation energy was an important factor affecting VRR (14). In our study, the ablation energy was also closely associated with the volume reduction of PTMC lesion after MWA. In addition, VRR also varies with ablation techniques. A meta-analysis showed that the average VRR of radiofrequency ablation (99.3%) was higher than those of MWA (95.3%) and laser ablation (88.6%) (15).

So far, cellular morphological changes of PTMC after thermal ablation have been rarely reported. After thermal ablation are processes of aseptic inflammation, fibrosis of necrotic tissue, and absorption of necrotic material. We observed histological features after MWA, including tissue damage (such as hemorrhage), degeneration and necrosis (such as acellular hyalinization, infarction), and repair (such as fibroblast proliferation, cholesterol granuloma). Ha et al. (16) demonstrated that hyaline degeneration was the most common pathological manifestation of benign nodules after MWA. In this study, fibroblast proliferation was the most frequent, while hyaline degeneration was occasionally detected, which may be attributed to the potential of papillary carcinoma to induce the proliferation of collagen fibers. Chronic inflammation is detected in both benign nodules and PTMC after ablations, which may be related to thermal damage. MWA did not immediately alter the follicular architecture of the thyroid tissue adjacent to the coagulation zone or the tumor capsule and induce fibrosis, without signs of cellular damage, extrafollicular colloid spread, inflammatory reaction, which may be due to undertreat similar to that seen in benign thyroid nodules (16, 17).

Thermal ablation may induce complications, including neck subcutaneous hematoma, fever, severe pain, voice changes, skin burns, edema, and hypothyroidism, or even permanent vocal cord paralysis, brachial plexus injury and nodule rupture in severe cases. However, our study proved that the incidence of complications was very low, and none developed permanent complications. MWA might bring about more complications than others, including short-term hoarseness, burning sensation, choking and coughing, toothache, slight changes in voice, discomfort in the anterior cervical area, and neck swelling, but all were mild (18). Compared with surgical treatment, incidences of transient or permanent hypothyroidism, transient hypoparathyroidism, hoarseness and dysphagia after MWA are significantly reduced. Notably, the lengths of stay and MWA operation were significantly lower than those of surgical treatment (9, 19, 20). In our study, no severe and permanent complications occurred after MWA. The pain was the most common but tolerable, without need of intervention, and usually disappeared within 1 week. One day after MWA, TSH was significantly reduced, whilst FT3, FT4 and Tg were remarkably elevated, a change that may be correlated to releases of thyroxine and Tg into the blood after ablation of thyroid tissues. Most PTMC patients developed euthyroidism within 1 month after MWA. However, thyroid function degenerated in 4.7% patients at 6 months of follow-up with a preoperative normal level, which may be associated with thyroid antibodies and the preoperative use of iodinated contrast media, since it can increase the risk of thyroid function disorder (21). At the meantime, thyroid dysfunction could present at any time after ablation, even 6 months later. Therefore, thyroid function should be carefully monitored after MWA.

We admit there are some limitations in this study. First, this was a retrospective study with a small sample size carried out in a single center. Second, the follow-up period was not long enough to assess the long-term efficacy and recurrence rate. Third, a significant association between effectiveness and pathological changes was not found in this study. Further well-designed, large, and long-term studies are required to confirm the predictive value of pathological changes in the evaluation of effectiveness and recurrence rate.

## Conclusion

MWA under ultrasound guidance is effective and safe in the first-line treatment of low-risk PTMC. Ablation energy may be an essential factor for complete absorption rate and VRR. Fibrous tissue hyperplasia is the most common pathological change after MWA. Notably, this is a single-center study with a small sample size and a short-period follow-up. In the future, the efficacy of MWA in the treatment of low-risk PTMC should be validated in multi-center studies with a larger sample size and a longer follow-up.

## Data Availability Statement

The original contributions presented in the study are included in the article/supplementary material. Further inquiries can be directed to the corresponding authors.

## Ethics Statement

The studies involving human participants were reviewed and approved by The ethics committee of Affiliated Hospital of Integrated Traditional Chinese and Western Medicine, Nanjing University of Chinese Medicine. The patients/participants provided their written informed consent to participate in this study. Written informed consent was obtained from the individual(s) for the publication of any potentially identifiable images or data included in this article.

## Author Contributions

CLu and XL developed the research questionnaire and wrote the protocol for this study. SX and CLi were responsible for the original study design and data collection together with the other authors. CLu and GC analyzed the data. YW and YX participated the treatment procedure of MWA. CLu, RL, JL, JW, and SX interpreted the results. CLu and XL wrote the article and the other authors revised it critically for important intellectual content. All authors contributed to the article and approved the submitted version.

## Funding

Design and data collection were supported by the grant supports of Jiangsu Provincial Key Research and Development Program (BE2020726) and the Leading Talents of Traditional Chinese Medicine in Jiangsu Province (SLJ0209).

## Conflict of Interest

The authors declare that the research was conducted in the absence of any commercial or financial relationships that could be construed as a potential conflict of interest.

## Publisher’s Note

All claims expressed in this article are solely those of the authors and do not necessarily represent those of their affiliated organizations, or those of the publisher, the editors and the reviewers. Any product that may be evaluated in this article, or claim that may be made by its manufacturer, is not guaranteed or endorsed by the publisher.
